# Population Pharmacokinetic Analysis of Tiropramide in Healthy Korean Subjects

**DOI:** 10.3390/pharmaceutics12040374

**Published:** 2020-04-18

**Authors:** Seung-Hyun Jeong, Ji-Hun Jang, Hea-Young Cho, Yong-Bok Lee

**Affiliations:** 1 College of Pharmacy, Chonnam National University, 77 Yongbong-ro, Buk-Gu, Gwangju 61186, Korea; rhdqn95@naver.com (S.-H.J.); jangji0121@naver.com (J.-H.J.); 2 College of Pharmacy, CHA University, 335 Pangyo-ro, Bundang-gu, Seongnam-si, Gyeonggi-Do 13488, Korea; hycho@cha.ac.kr

**Keywords:** tiropramide, healthy Korean subjects, modeling, population pharmacokinetic

## Abstract

The purpose of this study was to perform population pharmacokinetic (PPK) analysis of tiropramide in healthy Korean subjects, as well as to investigate the possible effects of various covariates on pharmacokinetic (PK) parameters of tiropramide. Although tiropramide is commonly used in digestive system-related diseases as an antispasmodic, PPK reporting and factors affecting PKs are not clearly reported. Thus, this study for healthy subjects is very significant because it could find new covariates in patients that had not been reported before or predict PPK for patients in the clinic by establishing PPK in healthy adults. By using Phoenix NLME, PK, demographic, and genetic data (collected to explain PK diversity of tiropramide in population) analyses were performed. As a basic model, a one-compartment with first-order absorption and lag-time was established and extended to include covariates that influenced the inter-subject variability. The total protein significantly influenced the distribution volume and systemic clearance of tiropramide, but genetic factors such as *ABCB1* (1236C>T, 2677G>T/A, and 3435C>T), *CYP2D6* (*1 and *10), *OCT2* (808G>T), and *PEPT1* (1287G>C) genes did not show any significant association with PK parameters of tiropramide. The final PPK model of tiropramide was validated, and suggested that some of the PK diversity in the population could be explained. Herein, we first describe the establishment of the PPK model of tiropramide for healthy Korean subjects, which may be useful as a dosing algorithm for the diseased population.

## 1. Introduction

Tiropramide is a drug that has been used from the past to the present for symptom relief and treatment of diseases of the digestive system such as acute spastic abdominal pain and irritable bowel syndrome. Tiropramide is a structurally equivalent tyrosine derivative and contains amide functional groups in the structure together with tertiary amines. It has been reported that tiropramide is widely metabolized into various metabolites (hydroxytiropramide, *N*-despropyltiropramide, *N*-desethyltiropramide, and *N*-desethyl-*N*-despropyltiropramide) after oral administration to rats and humans [1,2,3]. The mechanism of action of tiropramide has been reported to directly affect the smooth muscle cells. That is, tiropramide activates intracellular cyclic adenosine monophosphate (cAMP) synthase (adenylcyclase) to increase the amount of cAMP to regulate calcium ions necessary for muscle contraction, thereby controlling the relaxation and contraction of intestinal smooth muscle cells. This mechanism of action is very important for clinical use. Other antispasmodics include anticholinergics or antimuscarinics that relieve abdominal pain by acting on the intestinal nervous system. However, the mechanism of action on the nervous system may cause systemic side effects such as dry mouth, dry eyes, and drowsiness, which may lower the patients’ compliance with medication. As a result, tiropramide has been widely used in the clinic for the treatment of diseases related to the digestive system with the reduction of the systemic side effects.

However, reporting of pharmacokinetic (PK) information of tiropramide for humans is still very limited. Above all, tiropramide’s population pharmacokinetics (PPK) have not been reported thus far. Tiropramide has been reported to have hypotension and peripheral vasodilation (as side effects) like other anticholinergic or antimuscarinic drugs only when a high dose is administered. Overall, incidences of tiropramide side effects are very low and a very safe profile in humans has been reported [4]. Although the incidence of adverse effects of tiropramide is reported to be low in humans, optimal dosing algorithm may maximize the therapeutic effect of the drug and reduce its adverse effects by using a PPK model. PPK modeling can enable effective dose setting and individualized pharmacotherapy by quantifying the diversity of the drug concentrations among individuals (in the population) with a variety of related physicochemical factors. In addition, identifying the physicochemical factors affecting PKs of tiropramide will be a significant scientific basis for the clinical application (such as usage and dose settings) and formulation of tiropramide in the future.

Tiropramide is usually administered orally to adults at 100 mg (1 tablet) 2-3 times a day in the clinic. In exceptional cases, it is reported that tiropramide may be additionally administered in cases of less symptomatic relief. However, it is difficult to judge whether these levels of capacity and usage are precise when considering the differences among individuals in the population. More scientific evidence and data on the dose setting and safety of tiropramide are needed. Therefore, we thought that a study of the PPK model of tiropramide was necessary. In addition, it begs the question on how the various physicochemical or genetic factors among individuals in the population affect the PKs of tiropramide. Moreover, even if individual physicochemical or genetic factors affect tiropramide’s PKs, it is very doubtful as to how large the effect would be. Finding significant covariates of tiropramide is very difficult due to the lack of detailed PK information on the precise metabolic mechanisms, absorption, distribution, and excretion of tiropramide (especially in humans), and no PPK studies have been reported in the past. Therefore, in this study, we set up a variety of candidate covariates early in the study to establish factors that significantly influence the PK of tiropramide. The process was based on the physicochemical or physiological properties of tiropramide that have been reported to date.

Tiropramide has amine groups in its structure and will be basic at pH (about 7.4) in vivo on the basis of its pKa value (of 3.1) [4]. Therefore, the covariate effect was confirmed by genotyping the *OCT* gene, which is known to be related to the absorption, distribution, and excretion of various organic cations [5]. Tiropramide is a substance derived from the amino acid tyrosine [6]. Therefore, the genotyping of the *PEPT* gene, which is known to be involved in the absorption and distribution of the peptide drugs such as peptides and β-lactam antibiotics, was performed to confirm the covariate effect [7]. P-glycoprotein (P-gp) is a transporter with a broad range of substrate specificities of about 170 kDa and is known to be mainly involved in the efflux of neutral or cationic substances. In addition, *ABCB1* has been reported as a gene that encodes the P-gp. In this study, the covariate effect was confirmed by genotyping *ABCB1* [8]. In the past, the metabolism of tiropramide in the liver was examined [1], and the covariate effect was confirmed by genotyping metabolism-related *CYP2D6*. In particular, we focused on single nucleotide polymorphism (SNP) of the *CYP2D6* gene related to phase I metabolism (oxidation, reduction, hydrolysis, etc.) in the liver, and tried to confirm the correlation with PKs [9]. In addition, we collected information on various physicochemical factors (including general functional indicators of the kidney and liver) and sought to find the major covariates affecting PKs.

We report on PPK modeling of tiropramide in this study, together with factors affecting PK diversity in the population, which have not been reported yet. In the tiropramide final PPK model (of this study), we quantitatively reflected on the total protein, physicochemical, or genetic factors with differences between individuals, suggesting that scientific dose setting can be possible. In these aspects, for clinical applications, the tiropramide’s PPK model would be a great advantage. As a result, development of a tiropramide’s PPK model for use in healthy Korean subjects was the purpose of this study. The developed tiropramide’s PPK model is expected to be useful for determining a valuable dosing algorithm for tiropramide in healthy Korean subjects. In addition, the identification of factors affecting PK of tiropramide is expected to be of great help in related future studies.

## 2. Methods

### 2.1. Study Design

Samples obtained from a bioequivalence study of tiropramide in 24 healthy Korean males were included in this analysis. The age of these subjects ranged from 19 to 29 years (mean ± standard deviation (SD), 22.96 ± 2.61 years). Their body weights ranged from 55.2 to 82.8 kg (mean ± SD, 67.60 ± 7.28 kg). Their body surface area (BSA) ranged from 1.58 to 1.97 m^2^ (mean ± SD, 1.80 ± 0.11 m^2^), and their body mass index (BMI) ranged from 17.89 to 29.06 kg/m^2^ (mean ± SD, 22.74 ± 2.72 kg/m^2^). Each subject had no hypersensitivity to any drugs or previous history of illness and was physically normal. All subjects provided informed written consent to perform bioequivalence and PK studies. All subjects underwent physical examinations, clinical screening, complete blood count, urinalysis, and analysis of blood chemistry prior to the admission of this study to evaluate their physical health status. If subjects were taking any medications, other drugs, and/or alcohol for at least 1 week prior to this study, they were excluded from this study. The Institutional Review Board of the Institute of Bioequivalence and Bridging Study, Chonnam National University, Gwangju, Republic of Korea, reviewed and approved (Bioequivalence Test no. 875; 01.16.2003) this study protocol. Clinical studies were performed in accordance with rules of good clinical practice and the revised Declaration of Helsinki for biomedical research involving human subjects. Bioequivalence studies were performed as randomized, single-dose, open-label, crossover, and two-way studies. The data from reference formulation were only used for the current analysis. The subjects were hospitalized (Chonnam National University Hospital, Gwangju, Korea) at 7:30 p.m. 1 day before the study. The subjects had a heparin-locked catheter installed in the median cubital vein. A single dose (100 mg) of tiropramide was given orally to all subjects (after an overnight fast) in each study group with 240 mL of water. Before administration (0 h) and at 0.33, 0.67, 1, 1.5, 2, 2.5, 3, 4, 6, 8, and 12 h after oral administration, blood samples (8 mL) were collected into Vacutainer tubes. At the time of blood sampling, about 2 mL of blood was drained each time to completely remove the heparinized saline solution remaining in the IV catheter, and about 8 mL of blood was collected. Following centrifugation (for 20 min, at 5000 × *g*), plasma samples were obtained and transferred to polyethylene tubes. They were then stored at −80 °C until analysis. Another study was repeated in the same manner to complete the crossover design after a washout period of 7 days.

### 2.2. Determination of Physicochemical Parameters

Plasma samples were used in the determination of physicochemical parameters, including total proteins, aspartate transaminase (AST), albumin, alkaline phosphatase (ALP), alanine transaminase (ALT), and creatinine. The determination of main physicochemical parameters in this study, such as total proteins, albumin, AST, ALT, ALP, and creatinine, was performed in a dry automatic analyzer by microsides VITROS (Ortho Clinical Diagnostics, NJ, USA) operating by reflectance spectrophotometry.

### 2.3. Determination of Genotypes

The blood samples of 3 mL, obtained from individuals participating in this study, were used for genotyping. The blood samples were centrifuged for 10 min at 1000 × *g*, and deoxyribonucleic acid (DNA) was extracted from the leukocyte layer using a Wizard Genomic DNA purification kit (Promega Co., Madison, WI, USA). The extracted DNA was dissolved in 100 μL of DNA hydration solution and stored at −70 °C until analysis. In order to amplify a portion of gene containing a SNP by polymerase chain reaction (PCR) from the extracted DNA, each primer was prepared according to the variation of allele. Moreover, the genotypes were determined using appropriate methods that have been reported in the past [5,8,10,11]. We conducted experiments three times to acquire exact genotype data.

#### 2.3.1. Identification of ABCB1 1236C>T (in Exon 12), 2677G>T/A (in Exon 21), and 3435C>T (in Exon 26)

Candidate SNPs (1236C>T, 2677G>T/A, and 3435C>T) in the *ABCB1* gene were genotyped by using polymerase chain reaction–restriction fragment length polymorphism (PCR-RFLP). Each primer was prepared according to the variation of allele. Forward primer of 5′-TATCCTGTGTCTGTGAATTGCC-3′ and reverse primer of 5′-CCTGACTCACCACACACCAATG-3′ were used for *ABCB1* 1236C>T genotypes. Forward primer of 5′-TGCAGGCTATAGGTTCCAGG-3′ and reverse primer of 5′-TTTAGTTTGACTCACCTTCCCG-3′ were used for determination of *ABCB1* 2677G>T/A genotypes. Forward primer of 5′-TGTTTTCAGCTGCTTGATGG-3′ and reverse primer of 5′-AAGGCATGTATGTTGGCCTC-3′ were used for *ABCB1* 3435C>T genotypes. PCR was performed in 20 μL reaction mixture including 1 μL of 10 pmol each primer (forward and reverse), 1 μL extracted DNA, and 17 μL autoclaved distilled water. The PCR program was composed of an initial denaturation at 94 °C for 2 min followed by 35 cycles of denaturation at 94 °C for 30 s, annealing at 59.8 °C for 30 s, and extension at 72 °C for 30 s. The final extension step was performed at 72 °C for 5 min. The DNA fragments amplified by PCR were reacted at 37 °C for 16 h with restriction enzymes *HaeIII* (for *ABCB1* 1236C>T), *Sau3AI* (for *ABCB1* 3435C>T), and *BanI*/*RsaI* (for *ABCB1* 2677G>T/A), which can recognize and cleave specific sequences. These digested fragments were separated by electrophoresis in 2.5% agarose gel, and were visualized under ultraviolet light after staining the gel with ethidium bromide for 30 min.

#### 2.3.2. Identification of CYP2D6 Alleles

Polymorphisms (*1 and *10) in the *CYP2D6* gene of individuals were determined using DNA sequencing analysis. The primers were designed and prepared using the primer3 software. The PCR was performed in 20 μL reaction mixture including 1 μL extracted DNA, 1 μL of 10 pmol of each primer (forward and reverse), and 17 μL autoclaved distilled water. The PCR program was composed of an initial denaturation at 94 °C for 3 min followed by 35 cycles of denaturation at 94 °C for 20 s, annealing for at 62 °C 30 s, and extension at 72 °C for 20 min. A final extension step was conducted at 72 °C for 5 min. The PCR products were then analyzed by direct sequencing using the ABI PRISIM BigDye Terminator Cycle Sequencing Kit and an ABI Prism 3100 Genetic Analyzer (Applied Biosystems, Foster City, CA, USA).

#### 2.3.3. Identification of OCT2 808G>T

Candidate SNP (808G>T) in the *OCT2* gene was genotyped using pyrosequencing analysis. Each primer was prepared according to the variation of allele. Forward primer of 5′-CGGAGAACAGTGGGGATTTTTTAC-3′, reverse primer of 5′-CACGTAATTCCTTCCGTCTGAAGA-3′, and sequencing primer of 5′-GGTGGTTGCAGTTCACA-3′ were used for *OCT2* 808G>T genotype. Forward primer has a 5′ biotin triethylene glycol label necessary for post PCR processing. PCR was performed in 20 μL reaction mixture including 1 μL extracted DNA, 1 μL of 10 pmol each primer (forward and reverse), and 17 μL autoclaved distilled water. The PCR program comprised an initial denaturation at 94 °C for 5 min followed by 45 cycles of denaturation at 94 °C for 20 s, annealing at 52.1 °C for 30 s, and extension at 72 °C for 20 s. A final extension step was performed at 72 °C for 5 min. The biotinylated PCR products were immobilized on streptavidin-coated Sepharose beads (Amersham Biosciences, Uppsala, Sweden). A total of 37 μL of binding buffer (10 mM Tris HCl, 2 M sodium chloride, 1 mM EDTA, 0.1% Tween 20, pH 7.6; Pyrosequencing AB, Uppsala, Sweden), 3 μL of streptavidin-coated Sepharose beads, and 20 μL of water were added to 20 μL PCR product; then, the solution was vigorously shaken for 10 min at room temperature. A 96 pin magnetic tool (Pyrosequencing AB, Uppsala, Sweden) was used to transfer up to 96 samples at a time to solutions as follows. The beads with bound template were first transferred to 70% ethanol solution and 0.2 N sodium hydroxide solution, then to 1X washing buffer (Pyrosequencing AB, Uppsala, Sweden), and finally into a solution of 1X annealing buffer (20 mM Tris acetate, 2 mM magnesium acetate, pH 7.6), including the appropriate sequencing primer of 10 pmol. Lastly, this mixture was heated for 1 min to 95 °C and then cooled to 50 °C and incubated at room temperature for at least 5 min to bind the sequencing primer to the template. After template preparation, a 96-well plate including the samples was loaded into the instrument (PSQ 96MA; Pyrosequencing AB, Uppsala, Sweden) along with the optimal reagents (Pyro Gold; Biotage AB, Uppsala, Sweden). This instrument sequences the templates by dispensing reagents and deoxynucleotide triphosphates in a user-defined order, achieving real-time sequencing by synthesis in an automated fashion. This is achieved by creating and monitoring an enzyme cascade initiated by nucleotide incorporation that produces light emission. Pyrosequencing data were obtained by using Peak Height Determination Software v2.1 (Pyrosequencing AB, Uppsala, Sweden).

#### 2.3.4. Identification of PEPT1 1287G>C (in Exon 16)

Candidate SNP (1287G>C) in the *PEPT1* gene were genotyped by using PCR-RFLP. Each primer was prepared according to the variation of allele. Forward primer of 5′-CCCTTGTCAGGGTTAAGATGA-3′ and reverse primer of 5′-GCTTCTCTAAATCCTATTATAACAGGG-3′ were used for *PEPT1* 1287G>C genotypes. PCR was performed in 20 μL reaction mixture including 1 μL extracted DNA, 1 μL of 10 pmol each primer (forward and reverse), and 17 μL autoclaved distilled water. The PCR program comprised of an initial denaturation at 95 °C for 5 min followed by 35 cycles of denaturation at 95 °C for 20 s, annealing for at 54.5 °C 30 s, and an extension at 72 °C for 20 s. The final extension step was performed at 72 °C for 5 min. DNA fragments amplified by PCR were reacted at 37 °C for 1 h with restriction enzyme *Sau961*, which can recognize and cleave specific sequences. The digested fragments were separated by electrophoresis in 2.5% agarose gel, and were visualized under ultraviolet light after staining the gel with ethidium bromide for 30 min.

### 2.4. Determination of Plasma Tiropramide Concentrations

Plasma concentrations of tiropramide were determined using a validated column-switching semi-micro high-performance liquid chromatography (HPLC) method based on a previous study [12].

#### 2.4.1. Chromatographic Conditions

The analytical system consisted of the Nanospace SI-2 series (Shiseido, Tokyo, Japan) with an ultraviolet-visible (UV–VIS) detector 3002, two 3001 pumps, a 3014 column oven, a high pressure six-way switching valve 3012, a 3010 degasser, and a 3023 autosampler. The system operation and signal processing were operated by Syscon (Shiseido, Tokyo, Japan). The columns used in this on-line extraction system include an analytical column (Capcell Pak C_18_ UG120, 150 × 1.5 mm I.D. Shiseido), a pre-column (Capcell Pak MF Ph-1, 10 × 4 mm I.D. 5 μm Shiseido), and an enrichment column (Capcell Pak C_18_ UG120, 35 × 2 mm I.D. Shiseido). The mobile phase for primary separation of tiropramide in the pre-column and concentration in the enrichment column was phosphate buffer (50 mM, pH 7.0)-acetonitrile (88/12, v/v) with a flow rate of 0.5 mL/min. The mobile phase for analytical column was phosphoric acid–phosphate buffer (50 mM, pH 7.0)-acetonitrile (0.04/59.96/40, v/v/v) with a flow rate of 0.1 mL/min. All the columns were maintained at 25–30 °C. The quantification was performed at 230 nm wavelength. The peak with the retention time of tiropramide was verified using a photodiode array detector (2017 Diode Array, Shiseido, Tokyo, Japan).

#### 2.4.2. Analytical Procedures

The performance of column-switching semi-micro HPLC consists of three steps, as follows: sample loading and primary separation, enrichment of analyte fraction, and chromatographic separation. When the column-switching valve was at the precolumn inlet position, an aliquot of filtered (by 0.22 μm, Millex-GV syringe filter unit, Millipore, Burlington, MA, USA) plasma sample (80 μL) was loaded to the pre-column, and a primary separation of tiropramide from plasma proteins was conducted by using phosphate buffer (50 mM, pH 7.0)-acetonitrile (88/12, v/v). Subsequently, the valve was switched to the enrichment column inlet position, and the tiropramide fraction was eluted from the pre-column and concentrated in the enrichment column by phosphate buffer (50 mM, pH 7.0)-acetonitrile (88/12, v/v). Afterwards, the valve was switched to analytical column inlet position, and tiropramide was finally isolated and quantified by phosphoric acid–phosphate buffer (50 mM, pH 7.0)-acetonitrile (0.04/59.96/40, v/v/v).

### 2.5. Pharmacokinetic Analysis

Basic PK parameters of tiropramide were obtained from non-compartmental analysis (NCA) through the Phoenix-WinNonlin (8.1 version, Pharsight, Certara Inc., Princeton, NJ, USA) program. Peak plasma concentration (C_max_) and the time to reach C_max_ (T_max_) were individually determined using plasma concentration–time curve. The area under the curve (AUC_0–∞_) was calculated as the sum of AUC_0–t_ and C_last_/k, where C_last_ is the final measured concentration and k is the elimination rate constant at terminal phase. AUC_0–t_ was calculated using a linear trapezoidal rule from 0 to t (as 24) h after administration. The half-life (t_1/2_) was calculated as 0.693/k, and the volume of the distribution (V_d_/F) was calculated as dose/k·AUC_0–∞_. The clearance (CL/F) was calculated by dividing the dose of tiropramide by AUC_0–∞_, where F is the bioavailability of oral administration. All PK parameter values were calculated as mean ± SD, and the statistical differences in the group parameters (according to genotype) were confirmed by the analysis of variance (ANOVA) through the Statistical Package for the Social Sciences (SPSS) program (23 version, IBM). A *p*-value < 0.05 was established as being statistically significant.

### 2.6. Model Development

PPK analysis was conducted with a non-linear mixed effects model (NLME) approach through the Phoenix NLME (8.1 version, Pharsight, Certara Inc., Princeton, NJ, USA) program. In addition, PPK model development was performed in the first order conditional estimates method with extended least squares (FOCE-ELS) estimation (with *ŋ*–*ε* interaction).

As the first step of PPK modeling, data were fitted to two or one compartment disposition models with first order elimination and absorption kinetics without or with absorption lag-time for determining the structural base model (without covariates). In addition, a multiple transit model with a compartment added to the absorption phase was evaluated to establish the structural base model. The final selection of structural base model was performed by the statistical significance between models using goodness-of-fit (GOF) plots, twice the negative log likelihood (-2LL), and Akaike’s information criterion (AIC). The initial values for the parameters used in this process were obtained and referenced using NCA and classic compartment models. As a result, the basic PK parameters were as follows: clearance for the central compartment (CL), absorption lag time (T_lag_), volume of distribution for the central compartment (V), first oral absorption rate constant (K_a1_), and second oral absorption rate constant (K_a2_).

The residual variability was determined to additive error model in log transformed (plasma concentration) data, as shown in the following equation: C_obs,ij_ = C_pred,ij_·exp(ε_ij_), where ε_ij_ is the intra-subject variability (including model misspecification and assay error) with mean 0 and variance σ^2^, and C_pred,ij_ and C_obs,ij_ are the jth predicted and observed plasma concentrations in the ith subject, respectively.

The inter-individual variability (IIV) in PK parameters of tiropramide was evaluated by using an exponential error model, as shown in the following equation: P_i_ = P_tv_·exp(ŋ_i_), where ŋ_i_ is the random variable for the ith individual, which was normally distributed with mean 0 and variance ω^2^; P_i_ is the parameter value of the ith individual, and P_tv_ is the typical value of the population parameter.

As a second step of PPK modeling, candidate covariates (including demographic and genetic information) screened during this study were considered in reflecting the structural base model to account for PK diversity of tiropramide in the population. Height, body weight, age, BMI, BSA, creatinine, albumin, AST, ALT, ALP, creatinine clearance, and total protein were used as demographic candidate covariates. Here, BMI was determined by using the metric unit system [13]. BSA was determined on the basis of the Mosteller equation [14]. Creatinine clearance was determined on the basis of the Cockcroft–Gault equation [15]. There were also *ABCB1* 1236C>T, *ABCB1* 2677G>T/A, *ABCB1* 3435C>T, *CYP2D6* (*1 and *10), *OCT2* 808G>T, and *PEPT1* 1287G>C as genetic candidate covariates. To confirm the correlation between covariates and PK parameters, these potential covariates were plotted against individual post hoc parameters. In addition, the covariates were divided into categorical and continuous types in order to reflect the identified (correlation with PK parameters) candidate covariates in the PK parameters of the model. Continuous covariates (mainly demographic candidate covariates) were normalized by median values (of observed values). On the other hand, categorical covariates (mainly genetic candidate covariates) were reflected as index variables in the model. The effects of each covariate were confirmed using exponential, power, or additive options. By stepwise backward elimination and forward addition procedure, the covariates were included or eliminated. By change in the objective function value (OFV), the inclusion of covariates was determined. Covariates corresponding to a decrease in the OFV value greater than 3.84 (*p* < 0.05) were included in the base model (in the forward addition procedure). In addition, covariates corresponding to the case where the decrease in OFV value was greater than 6.63 (*p* < 0.01) through the backward elimination process were not removed from the model and were included.

### 2.7. Model Evaluation

The final established models (in this study) were verified and evaluated both visually and numerically. To evaluate the model, visual predictive check (VPC), bootstrapping, and goodness-of-fit (including distribution of residuals) analyses were used. The goodness-of-fit was confirmed by using diagnostic scatter plots as follows: (a) population-predicted concentrations (PRED) versus observed (DV), (b) individual-predicted concentrations (IPRED) versus DV, (c) PRED versus conditional weighted residuals (CWRES), (d) time (IVAR) versus CWRES, and (e) quantile–quantile plot of the components of CWRES.

By using non-parametric bootstrap analysis, the stability of the final model was confirmed, and the bootstrap option of Phoenix NLME was used. A total of 1000 replicates were generated by the repeated random sampling with replacement from the original dataset. The estimated parameter values, such as the standard errors (SE; including confidence intervals) and medians from the bootstrap procedure, were compared with those estimated from the original dataset.

By using the VPC option of Phoenix NLME, VPCs of the final established models were performed. The time–DV concentration data were graphically superimposed on the median values and the 5th and 95th percentiles of the time-simulated concentration profiles. If the DV concentration data were approximately distributed within the 95th and 5th prediction interval, the model was expected to be precise. Normalized prediction distribution error (NPDE) was used to evaluate the predictive performance of the model on the basis of a Monte Carlo simulation with the R package [16]. NPDE results were summarized graphically using (1) quantile–quantile plot of the NPDE, (2) a histogram of the NPDE, (3) scatterplot of NPDE vs. time, and (4) scatterplot of NPDE vs. PRED. If the predictive performance is satisfied, the NPDE will follow a normal distribution (Shapiro–Wilk test) with a mean value of zero (*t*-test) and a variance of one (Fisher’s test).

## 3. Results

### 3.1. Study Design and Demographic Analysis

The bioequivalence data (from reference formulation) collected from 24 healthy Korean males were used in this PK study for tiropramide. For the PK modelling, a total of 288 tiropramide plasma concentrations were available. There was complete information on height, age, and body weight for the 24 participants. Additionally, we successfully collected information on the total proteins, albumin, creatinine, AST, ALT, ALP, and the creatinine clearance levels of each participant, according to the method described above (Section 2.2). The related demographic information about the participants are shown in Table 1.

### 3.2. Genetic Analysis

Genotyping was performed on all 24 individuals who participated in this study. The analyzed genotypes were *ABCB1* (1236C>T, 2677G>T/A, and 3435C>T), *CYP2D6* (*1 and *10), *OCT2* (808G>T), and *PEPT1* (1287G>C) genes. The results are presented in Table 2. The *ABCB1* 1236C>T genotyping revealed that 6 (25.00%) subjects had the mutant type (TT), 13 (54.17%) subjects had the heterozygous type (CT), and 5 (20.83%) subjects had the homozygous wild type (CC). *ABCB1* 2677G>T/A genotyping revealed that eight (33.33%) subjects had the mutant type (TT or AT or TA or AA), nine (37.50%) subjects had the heterozygous type (GT or GA), and seven (29.17%) subjects had the homozygous wild type (GG). *ABCB1* 3435C>T genotyping revealed that 3 (12.50%) subjects had the mutant type (TT), 12 (50.00%) subjects had the heterozygous type (CT), and 9 (37.50%) subjects had the homozygous wild type (CC). *CYP2D6* genotyping revealed that 6 (25.00%) subjects had the homozygous *10 allele (*10/*10), 14 (58.33%) subjects had the heterozygous *10 allele (*1/*10), and 4 (16.67%) subjects had the homozygous *1 allele (*1/*1). CYP2D6*2, *4, *5, *14A/B, *36, and *47 alleles were not detected in any of the subjects of this study. We classified the *CYP2D6* genotypes into three groups to investigate the impact of *CYP2D6* genotypes on the PKs of tiropramide as follows: extensive metabolizers (EMs) (*1/*1), heterozygous intermediate metabolizers (IMs) (*1/*10), and homozygous IMs (*10/*10), on the basis of the reports of the difference in *CYP2D6* enzyme activity according to *CYP2D6* genotypes [9,17]. *OCT2* 808G>T genotyping revealed that 8 (33.33%) subjects had the heterozygous type (GT) and 16 (66.67%) subjects had the homozygous type (GG). *PEPT1* 1287G>C genotyping revealed that 3 (12.50%) subjects had the mutant type (CC), 2 (8.33%) subjects had the heterozygous type (GC), and 19 (79.17%) subjects had the homozygous wild type (GG).

### 3.3. Determination of Plasma Tiropramide Concentrations

After the oral administration of a 100 mg dose, plasma concentrations of tiropramide were determined by a column-switching semi-micro HPLC method (as mentioned in Section 2.4). This method analyzed tiropramide at a run time of 25 min per sample, enabling PK studies on tiropramide. The linearity of calibration curve for tiropramide was excellent (*r*^2^ = 0.99) in human plasma, ranging from 2 to 500 ng/mL. Moreover, the calculation formula of the calibration curve was as follows: *y* = 384.51*x* + 320.45 (*p* < 0.01), where *y* is the peak area of tiropramide and *x* is the concentration (ng/mL) of the tiropramide. In addition, the lower limit of quantitation (LLOQ) for tiropramide was as low as 2 ng/mL, and was sufficient for PK studies after the oral administration of tiropramide tablet to humans. This assay has been validated for specificity, accuracy, precision, and sensitivity in order to be applied to accurate PK studies. There were no significant interferences derived from system or endogenous substances peaks, and we confirmed the identical tiropramide peak spectrum with the diode array detector. Intra-batch (*n* = 5) accuracies for tiropramide ranged from 100.70% to 113.50% with precision (coefficient of variation, CV) of < 13.57%. Inter-batch (*n* = 5) accuracies for tiropramide ranged from 98.00% to 111.42% with precision (CV) of < 9.23%.

### 3.4. Pharmacokinetic (NCA) Analysis

**Figure 1:**
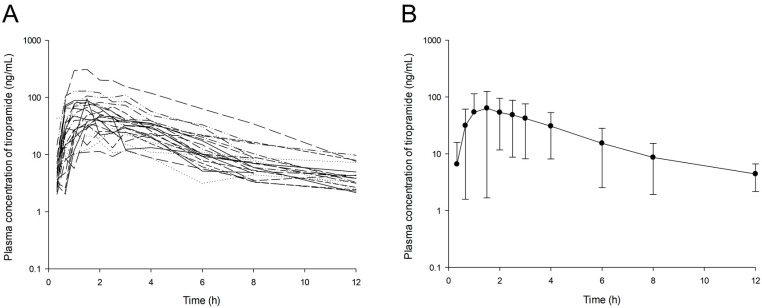
Log-transformed plasma concentration–time profiles of tiropramide in 24 subjects (**A**) and the mean curves (**B**). The vertical bars represent standard deviation of the mean.

The observed plasma concentration–time profiles of tiropramide in the 24 subjects after oral administration of the 100 mg dose are presented in Figure 1.

In most individuals, the concentration of tiropramide in blood was measured up to 12 h after dosing. The PK parameters of tiropramide according to the genotypes (*ABCB1* 1236C>T, *ABCB1* 2677G>T/A, *ABCB1* 3435C>T, *CYP2D6*, *OCT2* 808G>T, and *PEPT1* 1287G>C) calculated by NCA are presented in Table 3. According to the NCA results for each genotype, the diversity of PK parameters in each group was not significant (*p* > 0.05). Although the higher mean AUC_0-t_, AUC_0-∞_, and C_max_ values but lower mean CL/F values were calculated in the mutant type group (TT) than in the wild homozygous type groups (CC) in the *ABCB1* 1236C>T gene, this was not statistically significant considering the high SD. In the *CYP2D6* gene, higher mean AUC_0-t_, AUC_0-∞_, and C_max_ values were calculated for IMs (*1/*10 and *10/*10) than EMs (*1/*1), but this was also not statistically significant considering the high SD. In addition, although the higher mean C_max_, AUC_0-t_, and AUC_0-∞_ values and lower mean CL/F value were calculated in wild homozygous type groups (GG) than in mutant type group (CC) in the *PEPT1* 1287G>C gene, this was not statistically significant considering the high SD. The AUC_0-t_, AUC_0-∞_, C_max_, half-life, and T_max_ values calculated by the NCA analysis of tiropramide were 254.31 ± 197.38 h· ng/mL, 280.34 ± 199.96 h· ng/mL, 69.07 ± 59.74 ng/mL, 3.41 ± 1.99 h, and 1.74 ± 0.63 h, respectively.

### 3.5. Population Pharmacokinetic Model Development

**Figure 2:**
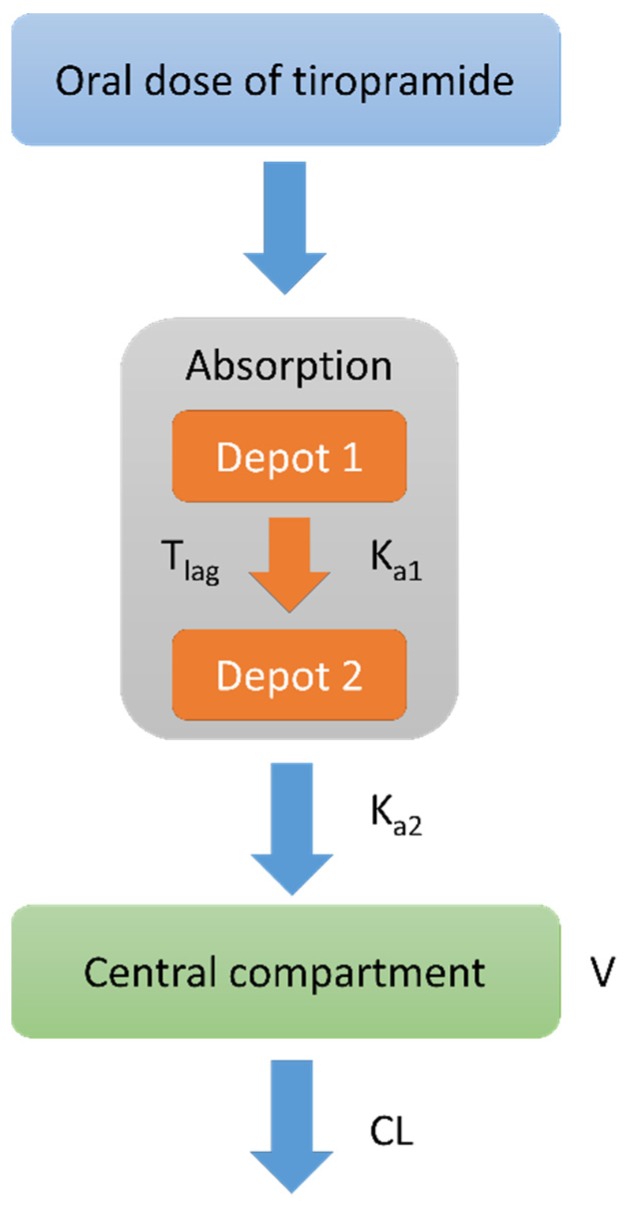
Schematic of tiropramide population pharmacokinetic (PPK) model (two transit absorption phase-one compartment model with an absorption lag time). Depot 1 and 2 represent transit compartments in the absorption phase. K_a1_ refers to the rate constant at which the drug is moved from depot 1 to depot 2. T_lag_ refers to the delay time for the drug to move from depot 1 to depot 2. K_a2_ is the rate constant at which the drug moves from depot 2 to the central compartment. V means the volume of drug distribution in the central compartment, and CL means removal of the drug from the central compartment.

By a one-compartment disposition model with first order elimination and absorption with an absorption lag time, the plasma concentrations of tiropramide were best expressed. Considering the lag time, the numerical values (such as -2LL and AIC) of the model evaluation and graphical data fitting were more improved than otherwise. Although we tried a two-compartment model, it did not show an improved fit when compared with the one-compartment model. In other words, there was a problem (fail to model fit) in fitting the two-compartment model for some individuals, but in the one-compartment model, all individuals were fitted properly. In addition, the -2LL and AIC values significantly decreased in the multiple (two) transit absorption compartment model in which the additional absorption phase was added to the one-compartment model. However, from two or more transit phases of absorption, there was no significant decrease in the -2LL and AIC values (with increasing number of parameters). As a result, the multiple (two) transit absorption phase-one compartment model with an absorption lag time was selected as the base structure model for tiropramide. The model was parameterized in terms of V/F, T_lag_, CL/F, K_a1_, and K_a2_. When the lag-time or model transit time was given to K_a2_ in the parameterization of the model, no significant model improvement was found with the increasing number of parameters. Therefore, significant model improvement was found when parameterization with lag-time was carried out only at K_a1_, and the rate constant of substance transit in the absorption compartment. Figure 2 shows a schematic of the disposition compartment model presented for tiropramide.

An exponential model was used to describe the IIVs on parameters of T_lag_, V/F, CL/F, K_a1_, and K_a2_. By applying an additive error model on log-transformed data, the residual variability was explained. Table 4 summarizes the steps for developing a basic structural model of tiropramide.

In order to find the covariates affecting the PK parameters of tiropramide, we analyzed the effects of each covariate on the PK parameters. The final potential covariates were selected on the basis of the graphical exploration between candidate covariates and PK parameters. The influence of each selected candidate covariate on the PK parameters of tiropramide was assessed by incorporating the covariates into an established basic structural model. The evaluation was based on OFV, which means model improvement. In this regard, the covariate selection process (according to OFV) to be reflected in the final model of tiropramide is summarized in Table 5.

**Figure 3:**
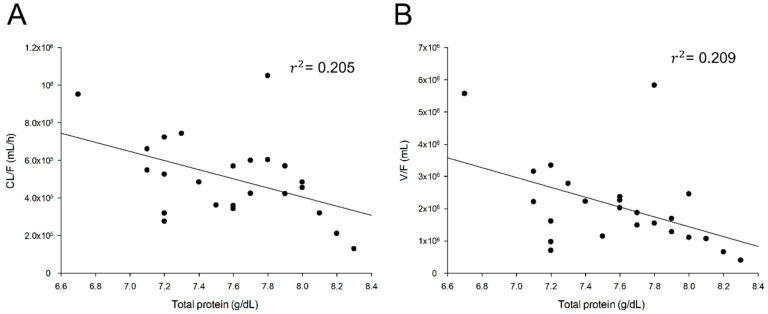
Relationship between subjects’ characteristics and individual predicted pharmacokinetic parameters. Clearance (CL/F) of tiropramide according to total protein (**A**). Volume of distribution (V/F) of tiropramide according to total protein (**B**).

There was a significant correlation between the total protein and tiropramide V/F as well as the total protein and tiropramide CL/F. Figure 3 shows the correlation between the final selected covariates and CL/F of tiropramide.

When the correlation between the total protein and CL/F was reflected in the PPK model of tiropramide, the ΔOFV was significantly reduced to −9.25 (*p* < 0.05). Furthermore, the addition of total protein and V/F correlation to tiropramide PPK model significantly reduced the ΔOFV to −9.16 (*p* < 0.05). However, other covariates including BMI, *PEPT1* 1287G>C, *OCT2* 808G>T, *ABCB1* 1236C>T, *ABCB1* 2677G>T/A, *ABCB1* 3435C>T, and *CYP2D6* (*1 and *10) had no significant effect on model improvement. Even by applying genetic factors (such as *PEPT1* 1287G>C, *OCT2* 808G>T, *ABCB1* 1236C>T, *ABCB1* 2677G>T/A, *ABCB1* 3435C>T, and *CYP2D6* (*1 and *10)) alone to the base model, we examined whether they affected K_a1_, K_a2_, CL/F, and V/F, but no significant associations were identified. The final model of tiropramide (reflecting the effects of covariates) is expressed as follows:

V/F = tvV/F · (1+ (Totalproteins-7.6) · dV/FdTotalproteins) · exp(*ŋ*_V_) CL/F = tvCL/F · (1+ (Totalproteins-7.6) · dCL/FdTotalproteins) · exp(*ŋ*_CL_) T_lag_ = tvT_lag_ · exp(*ŋ*_Tlag_) K_a1_ = tvK_a1_ · exp(*ŋ*_Ka1_) K_a2_ = tvK_a2_ · exp(*ŋ*_Ka2_) (1)

Population estimates of tiropramide were 466,711 mL/h for CL/F and 1,889,250 mL for V/F. CL/F and V/F values by NCA were 482,567 ± 267,433 mL/h and 2,386,871 ± 1,699,847 mL, respectively. As a result, the CL/F and V/F values estimated in the final model were not significantly different from the NCA values. In the final model, the relative standard error (RSE, %) was 9.53–83.51%. The Eta shrinkage values for the estimated PK parameters were suggested as acceptable at 0.02–0.40%. Compared with the base model, the final model (considering total protein effects) of tiropramide reduced the IIV of V/F from 70.70% to 57.12%, and the IIV of CL/F from 50.55% to 39.95%. Table 6 presents the estimated parameter values in the base model and final PPK model of tiropramide. The AUC_0–t_, AUC_0–∞_, C_max_, half-life, and T_max_ estimated values by the final PPK model of tiropramide were 242.73 ± 175.28 h· ng/mL, 260.92 ± 179.08 h· ng/mL, 54.89 ± 46.18 ng/mL, 2.73 ± 0.88 h, and 1.65 ± 0.53 h, respectively.

### 3.6. Population Pharmacokinetic Model Evaluation

**Figure 4:**
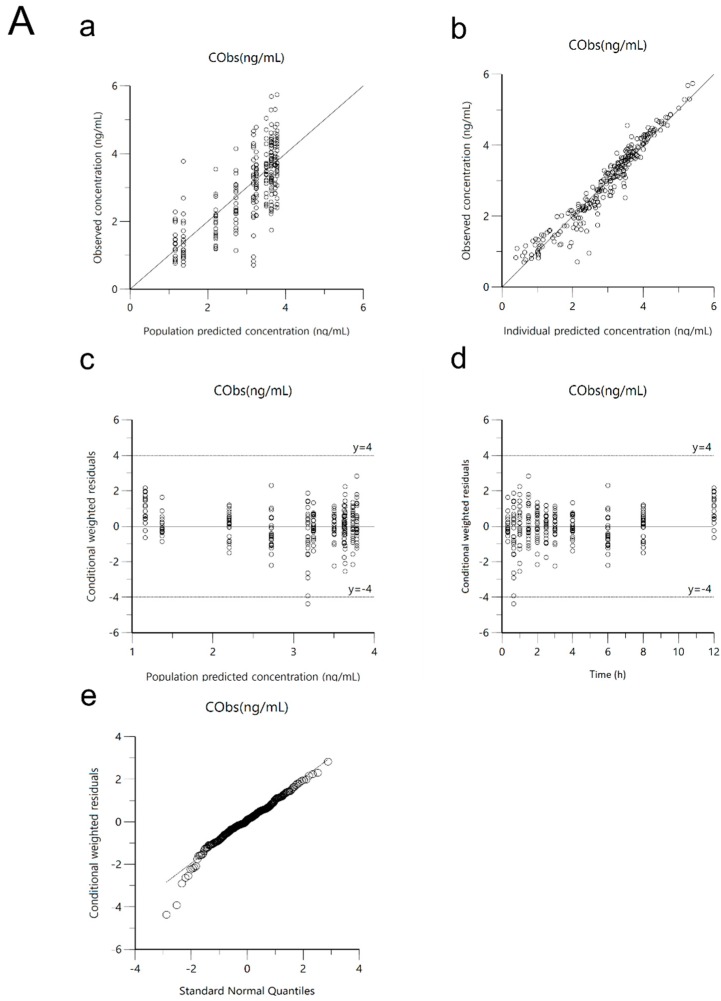

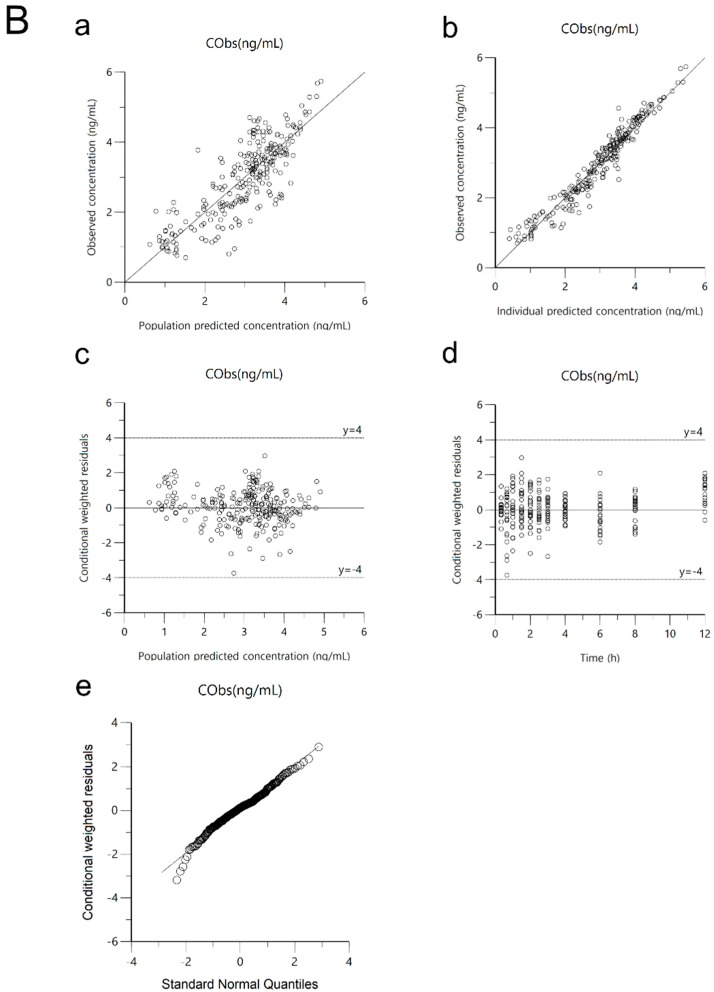
Goodness-of-fit (GOF) plots of base model (**A**) and final model (**B**) for tiropramide. (**a**) Population-predicted concentrations (PRED) against observed plasma concentration (DV), (**b**) individual-predicted concentrations (IPRED) against DV, (**c**) PRED against conditional weighted residuals (CWRES), (**d**) time (IVAR) against CWRES, and (**e**) quantile–quantile plot of components of CWRES.

The developed PPK model of the tiropramide was comprehensively evaluated for GOF, bootstrap analysis, and VPC. Figure 4 shows the GOF plots of the base and the final models of tiropramide. As shown in Figure 4B, the observed and predicted concentrations of tiropramide showed a relatively good agreement in the final model. CWRES was well distributed symmetrically with respect to zero, and CWRES was included in ±4 at all points. In addition, the residuals in the final model were more improved than in the base model. In other words, without any specific bias, the CWRES was randomly well distributed, and the residuals in the final model showed a significant decrease when compared with the base model (larger than ±4).

Bootstrap validation was performed to verify the reproducibility and/or robustness of the final PPK model of tiropramide. Table 7 shows the bootstrapping analysis results. The parameter values estimated in the final model were in the 95% CI range of the bootstrap analysis results, and were similar to the median values of the bootstrap (replicates of 1000).

**Figure 5:**
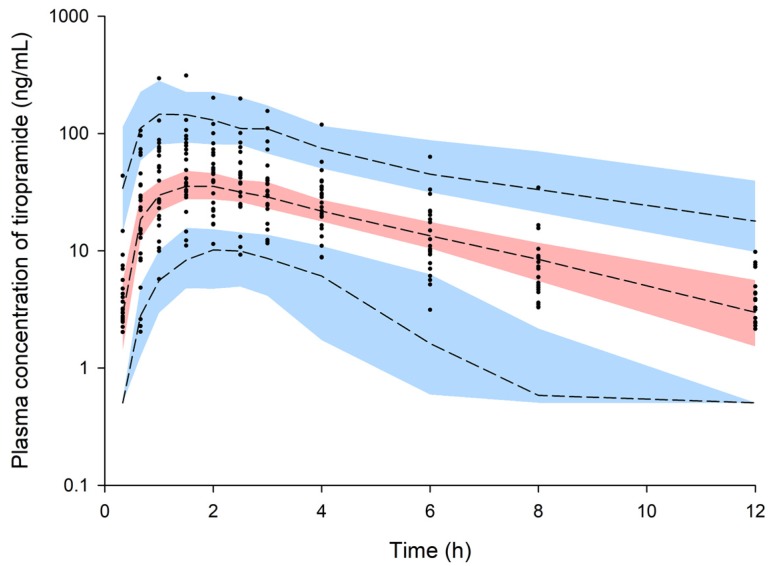
Visual predictive check (VPC) of the final model for tiropramide. Observed concentrations were depicted by the dots. The 95th, 50th, and 5th percentiles of the predicted concentrations are represented by black dashed lines. The 95% confidence intervals (CI) for the predicted 5th and 95th percentiles are represented by the blue shaded regions. The 95% CI for the predicted 50th percentiles are represented by the red shaded regions. The values on the *y*-axis are logarithms.

The VPC simulation results of the final PPK model of tiropramide are presented in Figure 5. Most of the observation values of tiropramide were well distributed within the 90% prediction interval of the prediction values. As a result, this suggests that the final model of the tiropramide is precise and explains the data well.

The NPDE distribution and histogram are presented in Figure S1. The assumption of a normal distribution for the differences between predictions and observations was acceptable. The quantile–quantile plots and histogram also confirmed the normality of the NPDE (Figure S1).

## 4. Discussion

The mechanism of action of tiropramide, as mentioned in the Introduction section, has been studied relatively well and has been reported in the past. However, studies on the in vivo PK characteristics (including metabolism and excretion) of tiropramide are still insufficient. Therefore, little data was available regarding the dosage and usage of tiropramide in clinical as well as formulation development. According to Lee et al., despite the frequent use of tiropramide in clinical practice, studies on safety and efficacy are very poor [4]. We conducted a PPK model development study of tiropramide to explore the effective covariates related to PK diversity of tiropramide and to investigate the characteristics of PK in the population. This study was new and was expected to be useful in the evaluation of the safety and efficacy of tiropramide in clinical use. As mentioned in the Abstract section, although tiropramide has a (relatively) broad margin of safety, this study involving healthy subjects was very important because it could find new covariates in healthy subjects that had not been reported before and/or be used to predict PPK for patients in the clinic by establishing PPK in healthy adults. In addition to this, in patients with abdominal pain and irritable bowel syndrome, it is very likely that the absorption process of tiropramide will change. Therefore, if clinical trials are conducted for patients in the future, it is thought that the PK variation of tiropramide between individuals can be explained more specifically through the application of our PPK model.

In this study, the PK of tiropramide was modelled as a two transit absorption phase-one compartment model with an absorption lag time. Various errors (including residual error and IIV) models and covariate effects were evaluated to establish factors that significantly influence the PK parameters of tiropramide and to explain the PK diversity of the tiropramide in the population. As a result of evaluating the model, the final tiropramide PPK model showed relatively good GOF plots, suggesting that the final PPK model had an acceptable predictive power. In addition, all CWRES values over time or predicted concentrations were in the range of -4 and 4, suggesting that the model is relatively stable. In addition, the bootstrap and VPC simulation results suggested that the final tiropramide PPK model was accurate, stable, and precise. We compared the estimated parameter values (of AUC_0–t_, AUC_0–∞_, C_max_, half-life, and T_max_) by tiropramide’s final PPK model with these values by NCA analysis. As a result, there were no significant differences (*p* > 0.05) between the parameter values predicted by the final PPK model of tiropramide and those calculated by NCA analysis. These results suggest that the final PPK model of tiropramide established in this study explains the experimental data relatively well.

The 24 healthy Korean male PK data used to establish the tiropramide PPK model were similar to the previously reported PK results. In other words, the previously reported PK parameter values of tiropramide were similar to our PK results obtained by NCA analysis. After oral administration of 100 mg of tiropramide in humans, the obtained NCA PK parameters (as previously reported values) were 2.34-6.99 h for t_1/2_, 0.66-1.6 h for T_max_, 77.4-111 ng/mL for C_max_, and 267.7-812.7 h·ng/mL for AUC [6,12,18,19,20,21]. On the other hand, the NCA PK parameters in this study were 3.41 ± 1.99 h for t_1/2_, 1.74 ± 0.63 h for T_max_, 69.07 ± 59.74 ng/mL for C_max_, and 280.34 ± 199.96 h·ng/mL for AUC, which were similar to the previously reported values. Table 8 summarizes these results.

As mentioned above, few studies have been done on the metabolic ratio (including pathway) and excretion of tiropramide in humans (especially patient groups), making it difficult to predict candidate covariates. On the basis of previous reports (as drug information provided by the manufacturer) that tiropramide is metabolized in the liver and excreted into urine (about 10–20% of administered dose), the covariate effects were tested in this study by obtaining the physicochemical information of liver function indicators (such as AST, ALT, and ALP) and genotyping the *CYP2D6* gene related to metabolism in the body. The creatinine clearance and the functional indicator of the kidney were collected for each subject, and the covariate effects related to CL/F were tested. In addition, genotyping of genes (such as *ABCB1*, *OCT2*, and *PEPT1*) associated with various transporters known to be widely involved in the distribution, absorption, excretion, and metabolism of drugs in the body has been performed to identify the effects of the covariate associated with the PK parameters. Despite these efforts, only total protein was found to have a significant effect on V/F and CL/F of tiropramide. As shown in Figure 3, the total protein, and the V/F and CL/F showed a significant negative correlation of 45.72% (*r*^2^ = 0.209) and 45.28% (*r*^2^ = 0.205), respectively. The correlation values of the total protein to CL/F and V/F were the largest of all the covariates we collected in this study.

Although the plasma protein binding ratio of tiropramide in humans has not been reported accurately, it has been reported that the plasma protein binding ratio of tiropramide in rats is about 48–51% [22]. This suggests that the amount of plasma protein may affect the in vivo PK properties of tiropramide by binding to the plasma proteins in the blood. According to our tiropramide PPK model, higher total protein levels in the blood mean a smaller distribution of tiropramide in the body and a lower excretion. This can be explained by the fact that tiropramide binds to proteins in the blood and affects the distribution and excretion of substances from the body. Tiropramide combined with proteins in the blood will make it difficult to filter the glomerulus of the kidney and distribute from blood to many organs. The reflection of the total protein covariates in tiropramide PPK model reduced V/F IIV and CL/F IIV by 13.58% and 10.60%, respectively. These results suggested that the variabilities of tiropramide plasma concentrations could be partly explained by individual variances of total protein level related with V/F and CL/F of tiropramide. On the other hand, other candidate covariates (such as AST, ALT, ALP, and creatinine clearance) had no significant effect on the PK parameter values and IIV improvement of tiropramide. Although tiropramide is metabolized in the liver and excreted in the kidney, our results suggest that tiropramide is a drug that does not require dose control depending on the liver and renal function. However, because our PPK model was based on the data from healthy men, further studies (for patient groups) will be needed for further clarification. That is, if PK data significantly different from the normal group (like our study) was obtained from the patient groups, and if the PPK analysis was conducted in the same manner as in this study with our model (from patient groups), other significant covariates may be identified. Therefore, PK studies or PPK analysis of tiropramide in patient groups will need to be performed in the future. Nevertheless, this study was important because it is a PPK model study of tiropramide that has not been previously conducted, and other related studies (such as clinical dose setting, formulation development, and PK comparison with certain other groups) on tiropramide may be possible in the future, on the basis of our findings. In addition, another limitation of our study was that PK analysis and PPK model studies were conducted for limited ages (between 19 and 29 years old). In the future, PK analysis and/or PPK model studies of tiropramide for more diverse age groups will need to be conducted in this regard.

As shown in Table 6, unexplained variability (as IIV) still exists in K_a_ (74.63% for K_a1_ and 74.57% for K_a2_) and T_lag_ (32.50%). These results meant that multi-complex gastrointestinal (GI) tract absorption processes of tiropramide including the variabilities of each individual gastric emptying time, GI tract transit time, and other transporters, among others, could considerably affect the variabilities of tiropramide plasma concentrations. This study could not find any significant covariates that could explain K_a_ IIV and T_lag_ IIV. Perhaps, despite collecting various genetic and demographic information, there was still not enough information to explain the K_a_ and T_lag_ IIVs of tiropramide. Therefore, further studies are needed to explore significant covariates related to the absorption of tiropramide in the body.

## 5. Conclusions

A PPK model for tiropramide was developed on the basis of PK data for healthy Korean men in this study. The plasma concentration profiles for tiropramide were described well by a two transit absorption phase-one compartment model with an absorption lag time. The total protein was identified by significant covariates of CL/F and V/F for tiropramide, and their correlation was finally reflected in the PPK model of tiropramide. On the other hand, no significant correlation was found between genetic information such as *ABCB1*, *CYP2D6*, *OCT2*, *PEPT1*, and the PK parameters of tiropramide. To the best of our knowledge, this is the first time a PPK model has been studied for tiropramide, and it is expected to be a valuable resource for future studies (such as in clinical use, as well as dose control and formulation development).

## Figures and Tables

**Table 1:** Demographic information of the studied subjects (*n* = 24).

Physicochemical	Median (Range)	Mean ± SD
Age (year)	23 (19–29)	22.96 ± 2.61
Weight (kg)	66.8 (52.5–82.8)	67.60 ± 7.28
Height (cm)	171.45 (164.3–185.4)	172.59 ± 5.85
BSA (m^2^) *	1.80 (1.58–1.97)	1.80 ± 0.11
BMI (kg/m^2^) **	22.63 (17.89–29.06)	22.74 ± 2.72
Albumin (g/dL)	4.6 (4.2–5.4)	4.64 ± 0.27
Total proteins (g/dL)	7.6 (6.7–8.3)	7.59 ± 0.41
ALT (U/L)	20.5 (8–55)	22.29 ± 11.67
AST (U/L)	20 (10–32)	20.88 ± 5.81
ALP (U/L)	76 (49–126)	81.83 ± 20.66
Creatinine clearance (mL/min) ***	118.49 (91.78–159.77)	122.88 ± 22.25
Creatinine (mg/dL)	0.9 (0.6–1.1)	0.91 ± 0.13

* Body surface area (BSA) was determined on the basis of the Monsteller equation as follows: $\sqrt{\left(\mathrm{height}\;\left(\mathrm{cm}\right)\times \;\mathrm{weight}\;\left(\mathrm{kg}\right)/3600\right)}$; ** body mass index (BMI) was calculated as follows: body weight (kg)/height^2^ (m^2^); *** creatinine clearance was determined on the basis of the Cockcroft–Gault equation as follows: [(140-age) × body weight (kg)]/[serum creatinine (mg/dL) × 72].

**Table 2:** Genetic information of the studied subjects (*n* = 24).

Genotypes		No. (Frequency)
*ABCB1*		
1236C>T (exon 12)	CC	5 (20.83%)
	CT	13 (54.17%)
	TT	6 (25.00%)
2677G>T/A (exon 21)	GG	7 (29.17%)
	GT, GA	9 (37.50%)
	TT, AT, TA, AA	8 (33.33%)
3435C>T (exon 26)	CC	9 (37.50%)
	CT	12 (50.00%)
	TT	3 (12.50%)
*CYP2D6*	*1/*1	4 (16.67%)
	*1/*10	14 (58.33%)
	*10/*10	6 (25.00%)
*OCT2*		
808G>T	GG	16 (66.67%)
	GT	8 (33.33%)
*PEPT1*		
1287G>C (exon 16)	GG	19 (79.17%)
	GC	2 (8.33%)
	CC	3 (12.50%)

**Table 3:** Pharmacokinetic parameters of tiropramide after a single oral administration of a 100 mg tiropramide tablet according to the genotypes (mean ± SD, *n* = 24).

Genotypes		Half-Life (h−1)	Tmax (h)	Cmax (ng/mL)	AUC0–t (h·ng/mL)	AUC0–∞ (h·ng/mL)	CL/F (L/h)
*ABCB1*							
1236C>T	CC (*n* = 5)	2.91 ± 1.42	1.90 ± 0.42	104.56 ± 117.25	394.51 ± 376.32	419.54 ± 376.69	380.72 ± 246.10
	CT (*n* = 13)	3.80 ± 2.41	1.67 ± 0.70	49.06 ± 23.81	182.00 ± 60.62	209.85 ± 72.09	551.43 ± 263.69
	TT (*n* = 6)	2.99 ± 1.36	1.75 ± 0.69	82.83 ± 39.09	293.01 ± 149.02	317.08 ± 154.99	419.44 ± 292.77
*ABCB1*							
2677G>T/A	GG (*n* = 7)	3.86 ± 3.11	1.38 ± 0.52	52.88 ± 15.94	214.50 ± 69.45	247.94 ± 76.58	439.10 ± 136.89
	GT (*n* = 9)	2.77 ± 0.89	2.06 ± 0.73	88.92 ± 89.16	323.04 ± 294.51	339.44 ± 296.97	481.39 ± 359.30
	TT, AT, TA, AA (*n* = 8)	3.75 ± 1.73	1.69 ± 0.46	60.89 ± 39.69	210.97 ± 122.69	242.22 ± 135.50	522.84 ± 259.46
*ABCB1*							
3435C>T	CC (*n* = 9)	2.82 ± 1.11	1.52 ± 0.46	78.29 ± 88.96	294.77 ± 291.67	317.13 ± 293.75	452.72 ± 201.28
	CT (*n* = 12)	3.44 ± 1.45	1.92 ± 0.70	63.79 ± 38.63	235.35 ± 131.42	259.88 ± 136.73	524.94 ± 338.71
	TT (*n* = 3)	5.10 ± 4.82	1.67 ± 0.76	62.51 ± 23.63	206.48 ± 14.18	251.82 ± 44.95	405.08 ± 67.07
*CYP2D6*							
	*1/*1 (*n* = 4)	4.74 ± 4.00	1.25 ± 0.29	63.24 ± 23.66	240.38 ± 75.18	278.97 ± 67.41	379.92 ± 118.76
	*1/*10 (*n* = 14)	3.14 ± 1.17	1.76 ± 0.59	66.73 ± 72.90	258.15 ± 238.14	280.90 ± 240.02	484.79 ± 223.74
	*10/*10 (*n* = 6)	3.17 ± 1.84	2.00 ± 0.77	78.41 ± 45.97	253.49 ± 168.34	279.97 ± 177.22	547.04 ± 420.10
*OCT2*							
808G>T	GG (*n* = 16)	3.62 ± 2.29	1.51 ± 0.53	60.42 ± 29.24	218.24 ± 89.40	247.77 ± 99.03	459.49 ± 166.01
	GT (*n* = 8)	3.00 ± 1.20	2.19 ± 0.59	86.37 ± 96.84	325.60 ± 319.70	345.50 ± 321.06	529.63 ± 414.56
*PEPT1*							
1287G>C	GG (*n* = 19)	3.07 ± 1.38	1.82 ± 0.65	62.40 ± 32.14	226.40 ± 109.97	248.21 ± 115.65	506.33 ± 278.61
	GC (*n* = 2)	6.36 ± 6.07	1.25 ± 0.35	181.48 ± 184.26	621.48 ± 609.37	688.85 ± 546.19	211.72 ± 167.87
	CC (*n* = 3)	3.63 ± 0.41	1.50 ± 0.50	36.36 ± 9.98	183.99 ± 65.49	211.54 ± 80.59	515.08 ± 168.25

**Table 4:** Basic structural model building steps.

Model	Description	n-Parameter	-2LL	AIC	Δ-2LL	ΔAIC	Compared with
Absorption model							
1	One compartment with first order (no T_lag_)	7	562.27	576.27			
2	One compartment with the first order (add T_lag_)	9	508.80	526.80	−53.48	−49.48	1
3 *	One compartment with the first order (add T_lag_ and additonal absorption transit phase one)	11	420.97	442.97	−87.83	−83.83	2
4	One compartment with the first order (add T_lag_ and additonal absorption transit phase two)	13	419.84	442.11	−1.13	−0.86	3
Residual error model							
3 *	Log additive	11	420.97	442.97			
5	Additive	11	1964.95	1986.95	1543.98	1543.98	3
6	Proportional	11	1860.33	1882.33	1439.36	1439.36	3
IIV model							
7	Remove IIV K_a1_	10	428.64	448.64	7.67	5.67	3
8	Remove IIV K_a2_	10	428.64	448.64	7.67	5.67	3
9	Remove IIV T_lag_	10	434.18	454.18	13.21	11.21	3
10	Remove IIV V/F	10	500.86	520.86	79.89	77.89	3
11	Remove IIV CL/F	10	534.10	554.10	113.13	111.13	3

* Selected model.

**Table 5:** Stepwise search for covariates.

Model	Description	OFV	ΔOFV	n-Parameter	Compared with
1	Base model	420.97		11	
2	Total protein on CL/F	411.72	−9.25	12	1
3 *	Total protein on CL/F and V/F	402.56	−9.16	13	2
4	Total protein on CL/F and V/F, BMI on K_a1_	400.61	−1.95	14	3
5	Total protein on CL/F and V/F, *PEPT1* (1287G>C) on K_a1_	402.57	0.01	14	3
6	Total protein on CL/F and V/F, *ABCB1* (1236C>T) on K_a1_	401.63	−0.93	14	3
7	Total protein on CL/F and V/F, *CYP2D6* (*1 and *10) on K_a2_	397.15	−5.42	15	3

* Selected final model.

**Table 6:** Population pharmacokinetic parameters for tiropramide in base model and final model.

Parameter	Estimate	SE	RSE (%)	Shrinkage (%)	IIV (%)
Base model					
tvK_a1_ (1/h)	3.160	0.511	16.143		
tvK_a2_ (1/h)	3.171	0.510	16.086		
tvV/F (mL)	1,717,060.491	262,434.751	15.284		
tvCL/F (mL/h)	447,163.154	48,667.242	10.884		
tvT_lag_ (h)	0.196	0.021	10.480		
ω^2^_V/F_	0.500	0.170	33.973	0.044	70.701
ω^2^_Cl/F_	0.255	0.103	40.328	0.016	50.546
ω^2^T_lag_	0.106	0.089	84.320	0.225	32.538
ω^2^_Ka1_	0.608	0.266	43.735	0.399	77.986
ω^2^_Ka2_	0.613	0.261	42.630	0.398	78.269
σ	0.354	0.041	11.673		
Final model					
tvK_a1_ (1/h)	3.187	0.538	16.872		
tvK_a2_ (1/h)	3.183	0.524	16.464		
tvV/F (mL)	1,889,250.002	289,606.633	15.329		
tvCL/F (mL/h)	466,711.101	44,476.962	9.530		
tvT_lag_ (h)	0.196	0.021	10.590		
dCl/FdTotalproteins	−0.804	0.296	36.791		
dV/FdTotalproteins	−1.049	0.232	22.118		
ω^2^_V/F_	0.326	0.158	48.369	0.068	57.118
ω^2^_Cl/F_	0.160	0.067	41.977	0.023	39.947
ω^2^T_lag_	0.107	0.089	83.508	0.225	32.503
ω^2^_Ka1_	0.557	0.276	49.584	0.405	74.627
ω^2^_Ka2_	0.556	0.269	48.406	0.405	74.566
σ	0.357	0.042	11.895		

**Table 7:** Estimated population pharmacokinetic parameter values of tiropramide and bootstrap validation (*n* = 1000).

Parameter	Final Model		Bootstrapping	
	Estimate	95% CI	Median	95% CI
tvK_a1_ (1/h)	3.187	2.128–4.246	3.187	2.332–4.733
tvK_a2_ (1/h)	3.183	2.151–4.215	3.183	2.273–4.345
tvV/F (mL)	1,889,250.002	1,318,770.124–2,459,730.256	1,889,250.000	1,397,156.042-2,491,555.125
tvCL/F (mL/h)	466,711.101	379,098.212–554,324.163	466,711.000	374,186.003–550,036.012
tvT_lag_ (h)	0.196	0.155–0.237	0.196	0.144–0.234
dCl/FdTotalproteins	–0.804	−1.387–(−0.221)	−0.804	−1.050–0.082
dV/FdTotalproteins	–1.049	−1.506–(−0.592)	−1.049	−1.222–0.246
ω^2^_V/F_	0.326	0.017–0.636	0.296	0.020–0.572
ω^2^_Cl/F_	0.160	0.028–0.291	0.134	0.023–0.245
ω^2^T_lag_	0.107	0.068–0.282	0.085	0.045–0.215
ω^2^_Ka1_	0.557	0.016–1.098	0.524	0.016–1.063
ω^2^_Ka2_	0.556	0.028–1.084	0.518	0.013–1.049
σ	0.357	0.273–0.441	0.363	0.276–0.428

**Table 8:** Previously reported pharmacokinetic (PK) parameter values of tiropramide obtained by non-compartmental analysis (NCA) analysis.

References	Subjects	PK Parameters				
		Half-life (h)	Tmax (h)	Cmax (ng/mL)	AUC_0-∞_ (h·ng/mL)	CL/F (L/h)
Kwon et al. (2003) [21]	Human (*n* = 2, 100 mg dose)	-	0.66–2	87.3–191.3	267.7–812.7	-
Kwon et al. (2003) [20]	Human (*n* = 16, 100 mg dose)	2.34–2.61	-	96.4 ± 51.6	380.8 ± 239.0	-
Lee et al. (2003) [6]	Human (*n* = 4, 100 mg dose)	2.7 ± 0.5	1.6 ± 0.6	77.4 ± 33.0	319 ± 147	-
Baek et al. (2003) [12]	Human (*n* = 14, 100 mg dose)	6.3 ± 2.0	1.6 ± 0.6	111 ± 62	377 ± 220	-
Jhee et al. (2006) [19]	Human (*n* = 18, 100 mg dose)	6.99 ± 0.83	1.33 ± 0.34	98.77 ± 30.23	434.39 ± 102.35	-
Imran et al. (2007) [18]	Human (*n* = 12, 100 mg dose)	3.3 ± 1.0	1.5 ± 0.2	105.35 ± 15.7	375.4 ± 76.7	-
In this study	Human (*n* = 24, 100 mg dose)	3.41 ± 1.99	1.74 ± 0.63	69.07 ± 59.74	280.34 ± 199.96	482.87 ± 267.25

## Supplementary Materials

The following are available online at https://www.mdpi.com/1999-4923/12/4/374/s1, Figure S1: Normalized prediction distribution error (NPDE) for the final model.
