# Supplementary material for: Population Pharmacokinetic Analysis of Tiropramide in Healthy Korean Subjects
Source: Pharmaceutics. 2020 Apr 18;12(4):374. doi: 10.3390/pharmaceutics12040374 (PMC7238185; doi:10.3390/pharmaceutics12040374)
Supplement: Supplementary file 1 [file pharmaceutics-12-00374-s001.pdf]

pharmaceutics

MDPI

Supplementary Materials: Population Pharmacokinetic Analysis of Tiropramide in Healthy Korean Subjects

Seung-Hyun Jeong, Ji-Hun Jang, Hea-Young Cho and Yong-Bok Lee *

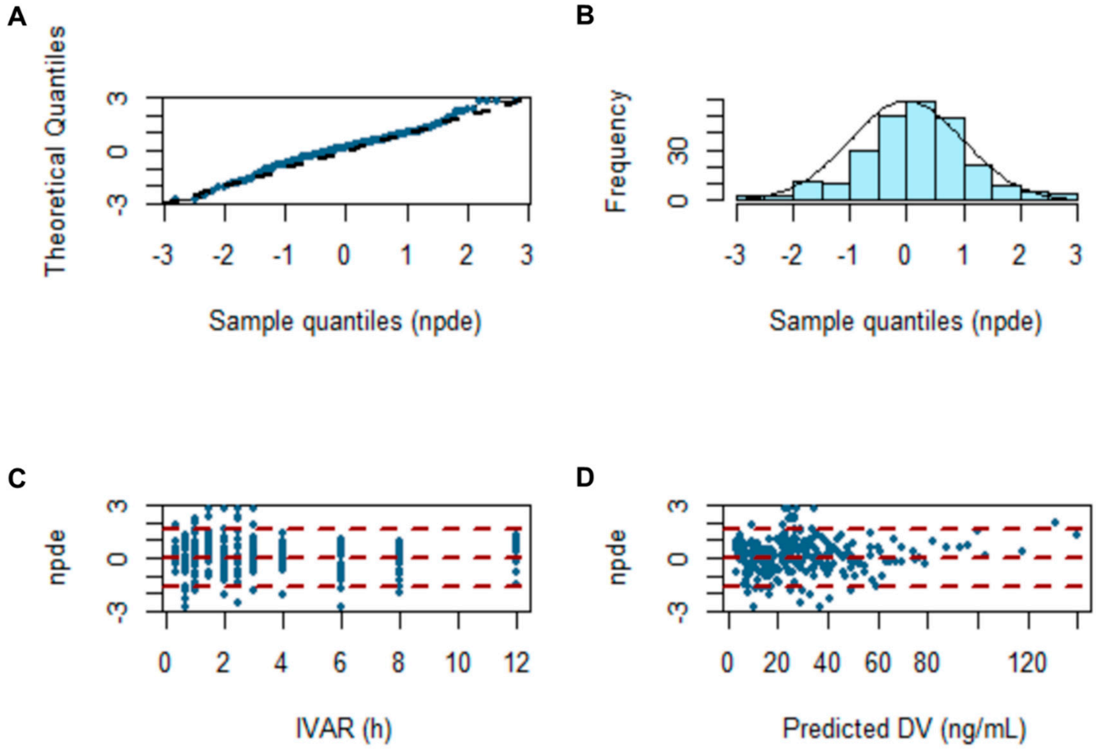

Figure S1. Normalized prediction distribution error (NPDE) for the final model. Quantile-quantile plots of NPDE versus the theoretical N (0, 1) distribution (**A**). Histogram the distribution of the NPDE, overlaid with the density of the standard Gaussian distribution (**B**). Scatter plot of the time versus NPDE (**C**). Scatterplot of predictions versus NPDE (**D**).
